# Gender Differences in Perceived Working Conditions of General Practitioners During the COVID-19 Pandemic—a Cross-Sectional Study

**DOI:** 10.1007/s11606-023-08166-8

**Published:** 2023-03-27

**Authors:** Dagmar Schaffler-Schaden, Lena Stöllinger, Alexander Avian, András Terebessy, Anna M. Scott, Sven Streit, Giuliano Piccoliori, Erika Zelko, Sebastian Huter, Karola Mergenthal, Herbert Bachler, Maria Flamm, Andrea Siebenhofer

**Affiliations:** 1 Institute for General Practice, Family Medicine and Preventive Medicine, Paracelsus Medical University Salzburg, Salzburg, Austria; 2 Institute for Medical Informatics, Statistics and Documentation, Medical University Graz, Auenbruggerplatz 2/5 8036, Graz, Austria; 3 Department of Public Health - Faculty of Medicine, Semmelweis University Budapest, Budapest, Hungary; 4 Institute for Evidence-Based Healthcare, Bond University Australia, Robina, Australia; 5 Institute of Primary Health Care (BIHAM), University of Bern, Bern, Switzerland; 6 Institute of General Practice, Institute for Special Training in General Medicine, Claudiana Bozen, Bolzano, Italy; 7 Faculty of Medicine Johannes, Kepler University of Linz, Linz, Austria; 8 Institute of General Practice, Goethe University Frankfurt, Frankfurt, Germany; 9 Institute of General Practice, Medical University Innsbruck, Innsbruck, Austria; 10 Institute for General Practice and Evidence Based Health Services Research, Medical University Graz, Graz, Austria

**Keywords:** Gender differences, General Practice, Pandemic, Self-Confidence, Risk-Perception

## Abstract

**Background:**

The ongoing COVID-19 pandemic has revealed gender-specific differences between general practitioners in adapting to the posed challenges. As primary care workforce is becoming increasingly female, in many countries, it is essential to take a closer look at gender-specific influences when the global health care system is confronted with a crisis.

**Objective:**

To explore gender-specific differences in the perceived working conditions and gender-specific differences in challenges facing GPs at the beginning of the COVID-19 pandemic in 2020.

**Design:**

Online survey in seven countries.

**Participants:**

2,602 GPs from seven countries (Austria, Australia, Switzerland, Germany, Hungary, Italy, Slovenia). Of the respondents, 44.4% (n = 1,155) were women.

**Main Measures:**

Online survey. We focused on gender-specific differences in general practitioners’ perceptions of working conditions at the beginning of the COVID-19 pandemic in 2020.

**Key Results:**

Female GPs rated their skills and self-confidence significantly lower than male GPs (f: 7.1, 95%CI: 6.9–7.3 vs. m: 7.6, 95%CI 7.4–7.8; p < .001), and their perceived risk (concerned about becoming infected or infecting others) higher than men (f: 5.7, 95%CI: 5.4–6.0 vs. m: 5.1, 95%CI: 4.8–5.5; p = .011). Among female GPs, low self-confidence in the treatment of COVID-19 patients appear to be common. Results were similar in all of the participating countries.

**Conclusions:**

Female and male GPs differed in terms of their self-confidence when dealing with COVID-19-related issues and their perceptions of the risks arising from the pandemic. To ensure optimal medical care, it is important that GPs realistically assess their own abilities and overall risk.

**Supplementary Information:**

The online version contains supplementary material available at 10.1007/s11606-023-08166-8.

## Introduction

The coronavirus pandemic has had a major impact on health systems worldwide. Managing the pandemic is a particular challenge for primary care.^1,2^ As many general practitioners (GP) had no previous experience of dealing with such a pandemic, it caught most of them unprepared and completely changed their work situation.^3^ For GPs, the psychological stress of responding to the pandemic was partly related to their role as the first point of contact for infected patients.^4^ Although several studies have described how GPs have dealt with the pandemic,^4,5^ findings concerning gender-specific differences between female and male GPs are scarce. Psychological side effects of the pandemic, such as depression, anxiety, and insomnia, particularly affect frontline female healthcare workers.^6,7^ Female health care workers are at higher risk of developing a Posttraumatic Stress Disorder during a pandemic, as reported in a recent systematic review on the coronavirus outbreaks of SARS, MERS, and COVID-19.^8^ Gender differences are also evident in medical education.

In 2021, 54.3% of first-year students in Australian medical schools were female.^9^ The proportion of female medical students in Germany (63.2%) is even higher.^10^ This trend is apparent in most western countries. Women are more likely to opt to specialize in a field in which it is easier to reconcile work and family life, such as general practice.^11^ Consequently, there is a predominance of female doctors in the primary care sector, which may have a significant impact on service supply (e.g. female work fewer hours and spend longer with their patients) in the future.^12^ In this context, it is worth mentioning that doctor-patient gender matching is generally preferred by patients and has beneficial health effects in primary care.^13^

Previous studies have shown that female medical professionals generally report having less confidence in their abilities and more anxiety than their male colleagues.^14,15^ This effect has been referred to as the “confidence gap” and can be found in all occupational groups and medical specialties.^16–18^ As in many fields of medicine, an inadequate risk assessment or excessively high or low self-confidence among GPs can have a direct impact on patient care. This issue is therefore particularly relevant in the management of a pandemic.

This study aimed to investigate gender-specific differences in the way GPs in seven countries coped with the challenges of working during the early phase of the COVID-19 pandemic. Besides other issues, we explored their self-confidence and their perceptions of risk when dealing with infected patients.

## Methods

The findings are reported in compliance with the CHERRIES checklist^19^ (Supplemental appendix 1). COVI-Prim-*Gender* is part of the international COVI-Prim project (Austria, Australia, Germany, Hungary, Italy, Switzerland, Slovenia) investigating the role of GPs during the COVID-19 pandemic.^1^ The COVI-Prim questionnaire^1^ consists of eight factors (preparedness for a pandemic, testing suspected cases, protection of staff, provision of information to GPs, perception of risk, self-confidence, decrease in number of patient contacts, efforts to control the spread of the disease; Supplemental appendix 2). Factor scores ranged from zero to ten. We transferred the questionnaire to LimeSurvey® (Austria, Germany, Hungary, Italy, Slovenia and Switzerland) and SurveyMonkey® (Australia). We invited GPs to respond to the questionnaire via participating universities in Austria, Australia, Germany, Hungary, Italy, Slovenia and Switzerland by using their respective mailing lists and local GP associations in Australia, Austria and Germany. In accordance with current data protection regulations, the study team had no direct access to the mailing lists. As the lists probably overlapped, it is impossible to know precisely how many GPs were contacted and to calculate a response rate. The first page of the survey provided information on its length, the investigators, and the purpose of the study, as well as consent information (Australia). After completion of the survey, all data on the online platform was stored in SPSS files. GPs received no incentive to participate. The survey ran from April 3 until August 4, 2020. A detailed description of the whole project (e.g. questionnaire development) is available elsewhere.^1^

### Statistics

Baseline characteristics are presented as mean (SD) or median (min–max), as appropriate. Categorical variables are provided as absolute numbers and in percent. In the main analysis, the influence of gender was controlled for environmental variables (country of survey: Germany vs. Austria; size of town of practice: < 5,000 vs. 5,000—< 20,000 vs. 20,000—< 100,000 vs. ≥ 100,000; role of the GP: employee vs. owner) and age, and analyzed using General Linear Models. Thus the main effects and two-way interactions of sex with other variables were included in the analysis. Estimated means and 95% confidence intervals were used to present the results. For a better understanding of results, significant results were described in more detail. When the results for a specific factor were significant, responses to the individual items it consisted of were also provided. In this presentation, the response categories “yes” and “probably yes” were combined, as were the response categories “probably no” and “no”. No statistical correction was carried out to adjust for non-representative samples. SPSS 26 were used for data analysis (IBM Corp 2019). A value of p < 0.05 was considered significant.

## Results

### Demographics

Overall, 2,602 GPs answered the survey [female: 44.4%, n = 1,155; age: 52.4 (9.9)]. Female GPs were significantly younger (p < 0.001). The percentage of female respondents differed between countries (p < 0.001) with the highest number in Slovenia (77.8%) and the lowest in Hungary (28.8%). More female than male GPs were employed (p < 0.001) and worked in bigger cities (p < 0.001). All demographic characteristics are provided in Table 1.

**Table 1:** Background and Sociodemographic Characteristics of GPs, Based on Responses to the COVI-Prim Questionnaire 2020 (n = 2,602)

	All	Male	Female
	n = 2,602	n = 1,447 (55.6%)	n = 1,155 (44.4%)
Age (years), mean (SD)	52.4 (9.9)	54.4 (9.8)	50.0 (9.6)
Country, n (%)			
Austria Australia Switzerland Germany Hungary Italy Slovenia	825 109 106 1268 156 75 63	510 (61.8%) 29 (26.6%) 72 (67.9%) 668 (52.7%) 111 (71.2%) 43 (57.3%) 14 (22.2%)	315 (38.2%) 80 (73.4%) 34 (32.1%) 600 (47.3%) 45 (28.8%) 32 (42.7%) 49 (77.8%)
Size of town of practice, n (%)			
< 5,000 5,000 – < 20,000 20,000 – < 100,000 ≥ 100,000	744 768 445 645	474 (63.7%) 446 (58.1%) 233 (52.4%) 294 (45.6%)	270 (36.3%) 322 (41.9%) 212 (47.6%) 351 (54.4%)
Position in the practice, n (%)			
Employee, locum etc Owner	391 2211	114 (29.2%) 1333 (60.3%)	277 (70.8%) 878 (39.7%)

### Overall Results

GPs gave low ratings on preparedness for a pandemic (3.6; 95% CI: 3.4–3.8), testing of suspected cases (3.8, 95%CI 3.6–3.9) and efforts to protect staff (2.4, 95%CI 2.2–2.5). The provision of information to GPs (4.9, 95%CI: 4.7–5.1), a decrease in patient contacts (5.7, 95%CI 5.5–5.9) and perceived risk (5.4, 95%CI 5.2–5.6) were rated as moderate. On the other hand, the participants rated their self-confidence (7.3, 95%CI 7.2–7.5), and their efforts to control the spread of the disease (7.2, 95%CI 7.0–7.3) highly. The distribution of all responses is given in Supplemental Appendix 3.

### Differences in Self-Confidence by Gender

**Fig. 1:**
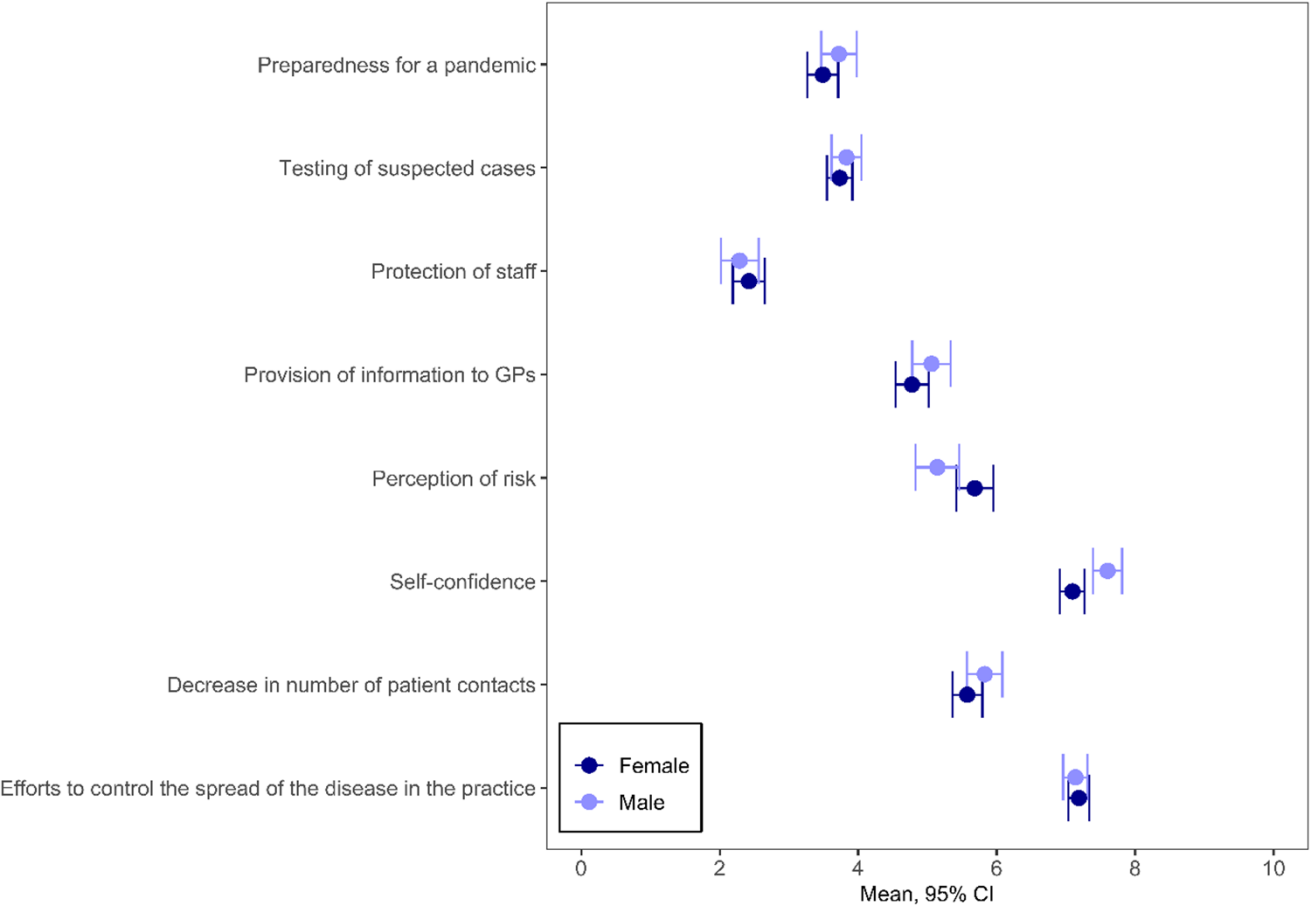
Differences between female and male GPs for the eight factors making up the COVI-Prim questionnaire

Female GPs rated their self-confidence lower than male GPs (7.1, 95%CI: 6.9–7.3 vs. 7.6, 95%CI 7.4–7.8; p < 0.001) (see Fig. 1). Looking at the individual items (Table 2) associated with self-confidence, a gender difference was observed in the proportion of GPs that were convinced they knew enough to provide optimal care for their patients during the pandemic (female: 78.6%, male: 84.1%; p < 0.001). Female GPs were more often unsure that they were doing everything right when caring for patients that had a COVID-19 infection (female: 40.1%, male: 26.4%, p < 0.001). Self-confidence increased with age for female GPs, but did not vary for male GPs (p = 0.01). The country, size of town and position in the practice had no identifiable influence on the relationship between sex and self-confidence.

**Table 2:**
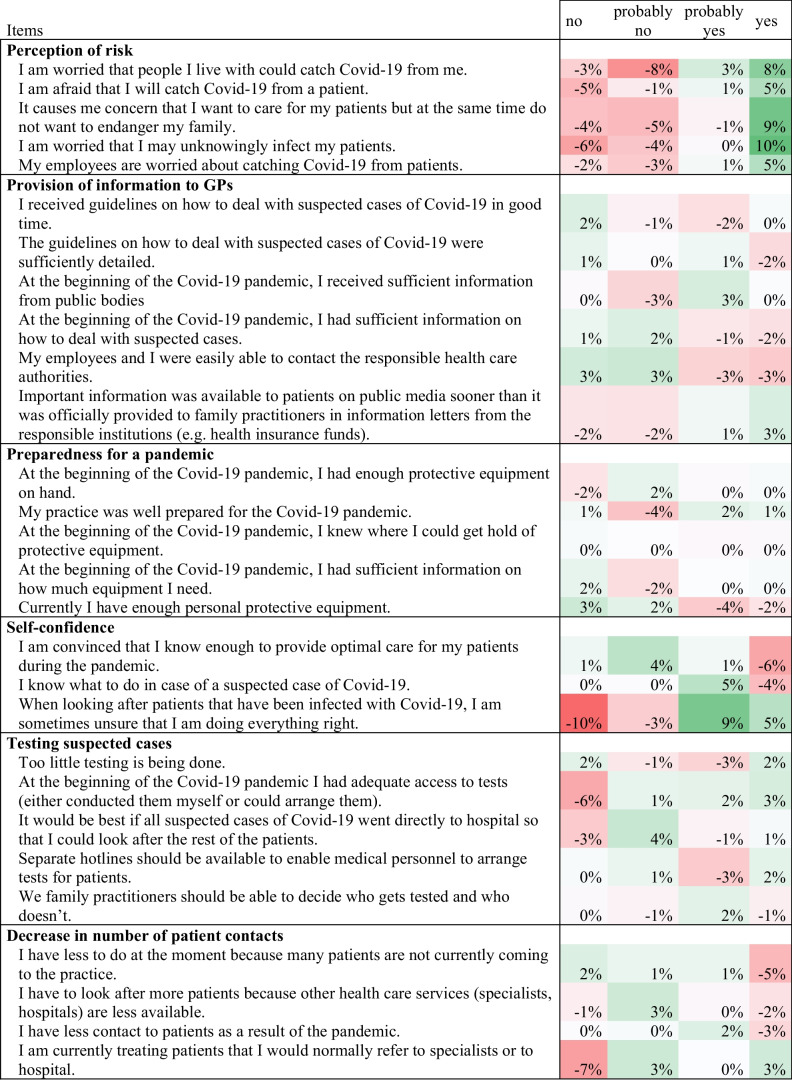

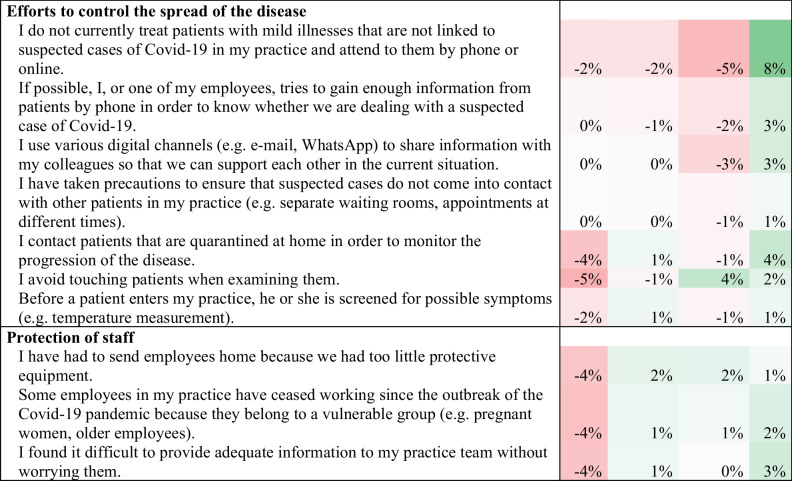
Difference in the responses to the COVI-Prim questionnaire 2020 of female (n = 1,155) and male GPs (n = 1,447). Percentages were calculated as %female GPs minus %male GPs. Responses which were more often chosen by female GPs are marked in green and responses which were more often chosen by male GPs are marked in red

### Differences in Perceived Risk Due to COVID-19 by Gender

Overall, female GPs rated their perceived risk due to COVID-19 higher (5.7, 95%CI: 5.4–6.0 vs. 5.1, 95%CI: 4.8–5.5; p = 0.011) than male GPs (Fig. 1). Among the individual items on the risk perception scale, the following gender differences were revealed (Table 2): More women were afraid of contracting COVID-19 from a patient (38.0% vs. 31.6%, p = 0.001), or of unknowingly infecting their patients (61.5% vs. 51.1%, p < 0.001). More female GPs reported that their employees were worried about contracting COVID-19 from patients (54.2% vs. 48.6%, p = 0.01). More female GPs were concerned about infecting people they lived with (63.4% vs. 52.6%, p < 0.001), and were concerned about putting their families at risk as a result of caring for patients with COVID-19 (58.6% vs. 49.5%, p < 0.001). Age, country, size of town and position in the practice had no significant influence on sex and perceived risk.

### Other Factors by Gender

There was no significant gender difference in the other factor scores, including preparedness for a pandemic, capacity for testing suspected cases, protection of staff, provision of information to GPs, decrease in number of patient contacts and efforts to control the spread of the disease. However, country-specific and position-specific gender effects could be observed. Male and female GPs rated the decrease in the number of patient contacts similarly in all countries except Italy (p = 0.04). In Italy, male GPs considered the decrease to be more pronounced (male: 6.0, 95%CI: 5.4–6.7; female: 4.3, 95%CI: 3.6–5.1).

Employed male GPs gave higher ratings than GP owners to both preparedness for a pandemic (employed: 4.1, 95%CI: 3.7–4.5; owners: 3.4, 95%CI: 3.1–3.6) and capacity for testing suspected cases (employed: 4.0, 95%CI: 3.6–4.3; owners: 3.7, 95%CI: 3.5–4.0). In contrast, there was no difference in preparedness for a pandemic between female GPs that were employed (3.5, 95%CI: 3.2–3.8) and practice owners (3.5, 95%CI: 3.3–3.7). Furthermore, testing is rated more highly by female owners (3.8, 95%CI: 3.6–4.0) than female employees (3.6, 95%CI: 3.4–3.9) (preparedness for a pandemic: p = 0.01; capacity of testing suspected cases: p = 0.03).

## Discussion

This cross-sectional study revealed that compared with their male colleagues, female GPs dealing with COVID-19-related issues had less self-confidence and perceived greater risk.

The lower self-confidence ratings and greater perceptions of risk among females were similar in all countries that participated in our survey. These findings are consistent with other previously published results. Differences in self-confidence between males and females are already apparent during medical studies. In her review article, Blanch-Hartigan reported as early as 2011 that male medical students tend to overestimate their abilities, while female students typically underestimate theirs.^20^ Later studies have also found that while female students reported lower confidence in their ability to perform physical examinations, diagnostic investigations, major interventions and other specific medical procedures (e.g. tooth and retained root extraction) than male students,^21,23^ this was not the case for minor interventions, their ability to interpret test results, basic patient assessments and “other skills”.^22^ A study by Witt et al. shows that these differences do not necessarily continue throughout their careers but can disappear as a result of training and as they gain experience.^24^ Although it may be tempting to assume that the self-assessments of male and female students have a factual basis and that female students are not only less confident, but also perform worse, studies have shown that the clinical performance of female students is actually of equal standard or better than that of their male colleagues.^25,26^

While many studies have examined differences in the self-confidence between male and female students, few studies have considered qualified medical doctors. In those that do exist, gender-specific differences in self-confidence can be found in both young and experienced doctors, as is the case with students. Male doctors express greater confidence than female doctors in their ability to perform physical examinations, interpret clinical tests, and carry out specific procedures (e.g. root canal treatment).^27,28^ No difference was found in terms of physician–patient relationships and their ability to perform social services.^28^ As in the case of students, training may help women that consider themselves to perform relatively poorly to ultimately have comparable confidence levels to men.^29^ Furthermore, as with students, the gender differences in self-confidence among physicians were not reflected in actual performance.^30,31^ Interestingly, Krautheim et al. found that for male ICU physicians, no association existed between self-confidence and the results of a knowledge test, whereas for female ICU physicians a weak association was present (r = 0.270).^32^

Considering that medicine is to some extent an uncertain science, it is important to understand the influence that confidence may have. Uncertainty is clinician-perceived and has, for example, the potential to influence diagnostic evaluations, and may result in diagnostic delays if improperly managed. It is also dynamic and evolves over time.^33^ Although greater experience and confidence generally make it easier to deal with uncertain situations in medicine, gender differences nonetheless exist.^34^ Faced with uncertainty, female physicians show higher stress reactions than males physicians (anxiety due to uncertainty, concern about bad outcomes) and differ in their strategies to overcome it.^35,36^ This may partly explain the differences in confidence between male and female GPs.

Problems in medical care that may result from overconfidence include, for example, diagnostic errors when a physician ceases to consider alternative diagnoses too early, or sees no need for further diagnostic tests.^37,38^ A mechanism that can reinforce poor decision-making in overconfident physicians is a lack of feedback or inadequate feedback. This is because patients may recover despite receiving an incorrect diagnosis, or may receive a correct diagnosis on returning with more pronounced symptoms. Furthermore, patients may respond to a drug that is not specific or selective, such as corticosteroids, even though the diagnosis is wrong.^37,39^ This lack of adequate feedback can lead to a self-confirming bias loop that increases confidence.^40^ According to Croskerry and Norman other sources of overconfidence in medicine include cognitive and affective bias, biased evidence-gathering, denial of uncertainty and a lack of critical thinking.^41^

Too little confidence may also have a negative impact on patients. In the worst case, such underconfidence in an uncertain situation may lead to an inability to decide what is best.^42^ As a result, further unnecessary examinations and tests are carried out, lengthening the diagnostic process.^43^ The extent to which additional training helps mitigate underconfidence is unclear. Kuhn et al. found that feedback made physicians more uncertain of themselves, resulting in an increase in underconfidence, while Nederhand et al. observed the opposite effect.^43,44^

As with confidence, how GPs feel about taking risks may have a significant impact on how clinical decisions are made. Attitudes towards risk and the perception of risk were found to influence hospital admission rates, the use of laboratory tests, the use of imaging in emergency departments, and the willingness to prescribe medications and update immunizations.^45–49^ It is therefore important to understand what influences medical doctors’ perception of risk. In addition to age, gender and personality, risk assessments may also be influenced by experience, own health behaviors, and profession (e.g. surgeons’ perception of risk is generally lower).^50–55^ With regard to COVID-19, several studies have shown that both women working in the medical field and female students working in medical and non-medical fields rated risk more highly.^56,57^ Differences in risk perceptions cannot be explained by differences in knowledge. As Licata et al. observed, gender does not affect students' knowledge of COVID-19, but it does affect risk perceptions and health behaviors.^58^ In this context, it is important that perceptions of risk correspond to reality and are neither too high nor too low. As Vancheri was able to show, the perceived risk of a patient developing coronary heart disease decreased in line with physician experience, leading to underestimates.^52^ Less experienced GPs, on the other hand, correctly assessed the risk more often than more experienced doctors. Differences in risk perceptions have also been identified in other non-medical fields. In this context, it is worthy of note that gender differences exist in emotional reactions to risky situations,^59^ and risk is considered more acceptable when decisions affect other people's outcomes.^60^ One explanation for risky behavior lies in overconfidence. Although in different experimental studies, both men and women were often overconfident in risky situations, men were generally more overconfident than women.^59^

Confidence and risk perceptions are linked both to uncertainty in the field of medicine and to one another. In both cases, it is important to assess a situation correctly. Excessive confidence is just as undesirable as too little confidence. Likewise, both the overestimation and underestimation of risk are undesirable. It is therefore important not simply to raise confidence levels among women, but to ensure as far as possible that confidence in men and women reflects reality. Uncertain situations are not only a consequence of the COVID-19 pandemic, but are typical of medicine, especially in the primary care setting. Therefore, this uncertainty and how to deal with it represents a challenge in daily practice and especially in the event of possible further pandemics.

### Strengths and Limitations

The main strengths of this study are the topicality of the data and the international approach. Furthermore, the role in the medical profession that is played by gender has rarely been addressed in academic medical research. This study therefore attempts to draw attention to a subject that has hitherto been neglected. However, as sociodemographic differences and disparities between minority groups were not explored, further research is needed to complement the findings of this study.

The main limitation of this study is that the questionnaire was developed in a relatively short time. Although GPs, psychometricians, psychologists and professional translators were included in its development, and extensive literature research was undertaken, it cannot be ruled out that some relevant aspects were not considered. Although little time was available for its development, the questionnaire was tested several times before being used.^1^

Furthermore, the period during which the survey was conducted was short and it only covers the first wave of the pandemic. Another limitation is that we cannot specify the response rate, as the GPs were contacted using different methods. As a result, some GPs may have been contacted several times and others not at all, so we do not know how many doctors were contacted in total. Data protection regulations prevented us from comparing the various lists of addresses.

## Conclusion

Female and male GPs differed in terms of their self-confidence when dealing with COVID-19-related issues and their perceptions of the risks arising from the pandemic. To ensure optimal medical care, it is important that GPs realistically assess their own abilities and overall risk. Both overconfidence and underconfidence, as well as the overestimation and underestimation of risk, can negatively influence health outcomes.

## Supplementary Information

Below is the link to the electronic supplementary material.

Supplementary file1 (DOCX 1580 KB)
