# Supplementary material for: Gender Differences in Perceived Working Conditions of General Practitioners During the COVID-19 Pandemic—a Cross-Sectional Study
Source: J Gen Intern Med. 2023 Mar 27;38(8):1894–901. doi: 10.1007/s11606-023-08166-8 (PMC10042103; doi:10.1007/s11606-023-08166-8)
Supplement: Supplementary file 1 — Supplementary file1 (DOCX 1580 KB) [file 11606_2023_8166_MOESM1_ESM.docx]

**Supplemental Digital Appendix**

**Supplemental Digital Appendix 1.** Checklist for Reporting Results of Internet E-Surveys (CHERRIES)

| 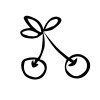 | **Checklist for Reporting Results of Internet E-Surveys (CHERRIES)** | |
| --- | --- | --- |
| ***Item Category*** | ***Checklist Item*** | ***Page*** |
| **Design** |  |  |
|  | Describe survey design | 4 |
| **IRB (Institutional Review Board) approval and informed consent process** |  |  |
|  | IRB approval | 5 |
|  | Informed consent | NA |
|  | Data protection | NA |
| **Development and pre-testing** |  |  |
|  | Development and testing | 4, Siebenhofer et al. 2021 |
| **Recruitment process and description of the sample having access to the questionnaire** |  |  |
|  | Open survey versus closed survey | Siebenhofer et al. 2021 |
|  | Contact mode | Siebenhofer et al. 2021 |
|  | Advertising the survey | Siebenhofer et al. 2021 |
| **Survey administration** |  |  |
|  | Web/E-mail | Siebenhofer et al. 2021 |
|  | Context | Siebenhofer et al. 2021 |
|  | Mandatory/voluntary | Siebenhofer et al. 2021 |
|  | Incentives | Siebenhofer et al. 2021 |
|  | Time/Date | Siebenhofer et al. 2021 |
|  | Randomization of items or questionnaires | Siebenhofer et al. 2021 |
|  | Adaptive questioning | Siebenhofer et al. 2021 |
|  | Number of Items | Siebenhofer et al. 2021 |
|  | Number of screens (pages) | Siebenhofer et al. 2021 |
|  | Completeness check | Siebenhofer et al. 2021 |
|  | Review step | Siebenhofer et al. 2021 |
| **Response rates** |  |  |
|  | Unique site visitor | NA |
|  | View rate (Ratio of unique survey visitors/unique site visitors) | NA |
|  | Participation rate (Ratio of unique visitors who agreed to participate/unique first survey page visitors) | NA |
|  | Completion rate (Ratio of users who finished the survey/users who agreed to participate) | Siebenhofer et al. 2021 |
| **Preventing multiple entries from the same individual** |  |  |
|  | Cookies used | Siebenhofer et al. 2021 |
|  | IP check | Siebenhofer et al. 2021 |
|  | Log file analysis | Siebenhofer et al. 2021 |
|  | Registration | NA |
| **Analysis** |  |  |
|  | Handling of incomplete questionnaires | Siebenhofer et al. 2021 |
|  | Questionnaires submitted with an atypical timestamp | Siebenhofer et al. 2021 |
|  | Statistical correction | 5 |

**Supplemental Digital Appendix 2.** COVI-Prim Questionnaire


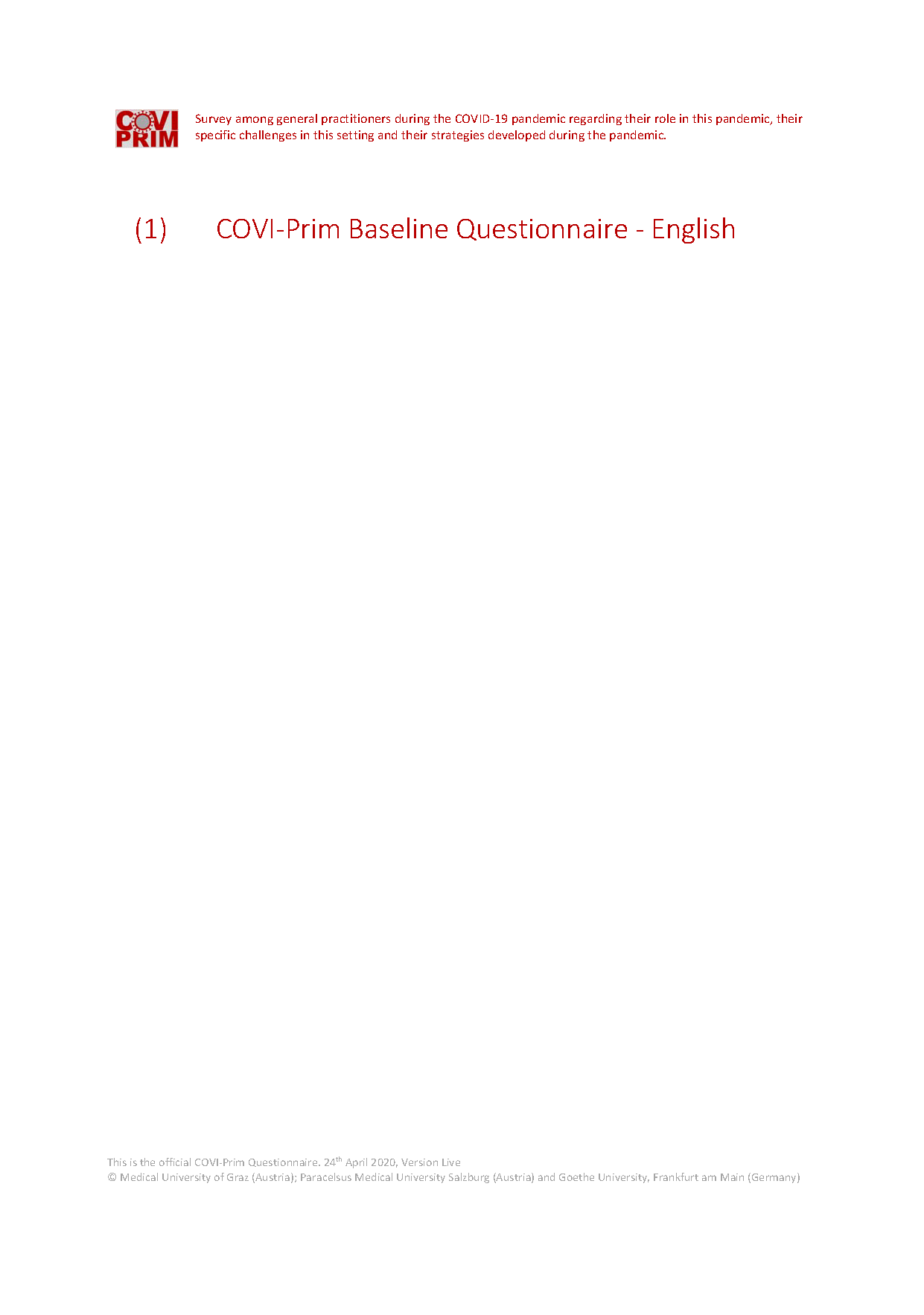


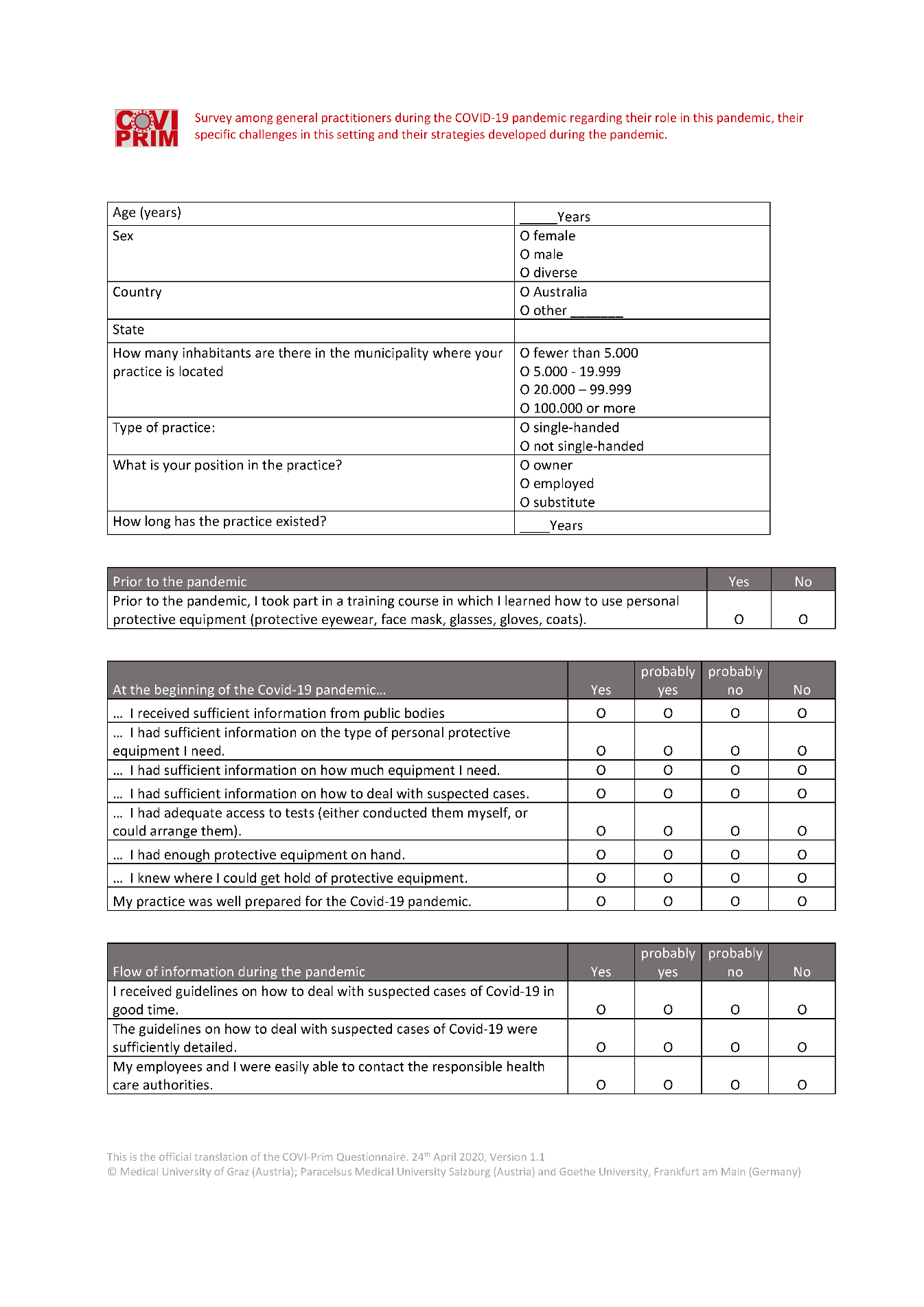

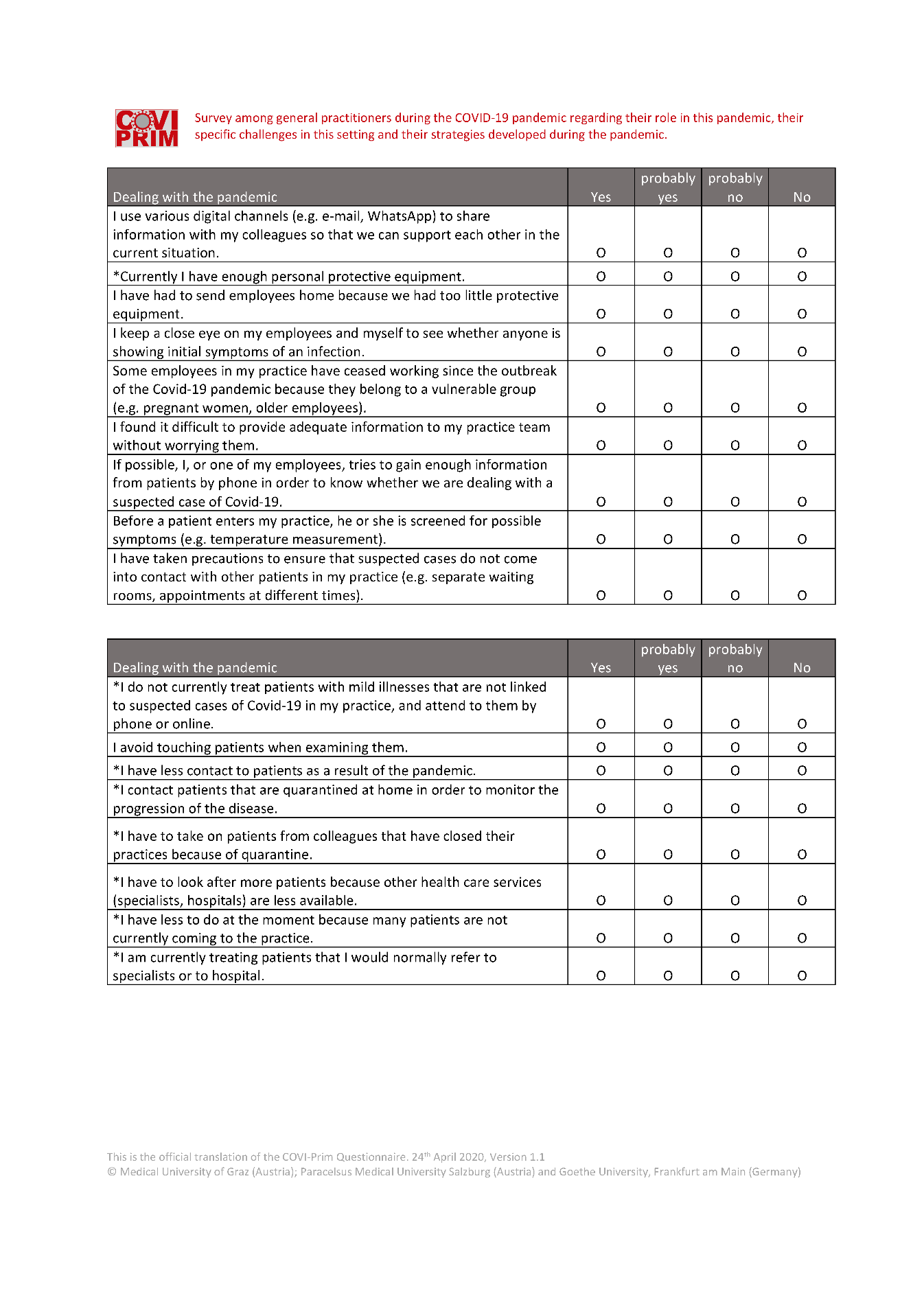

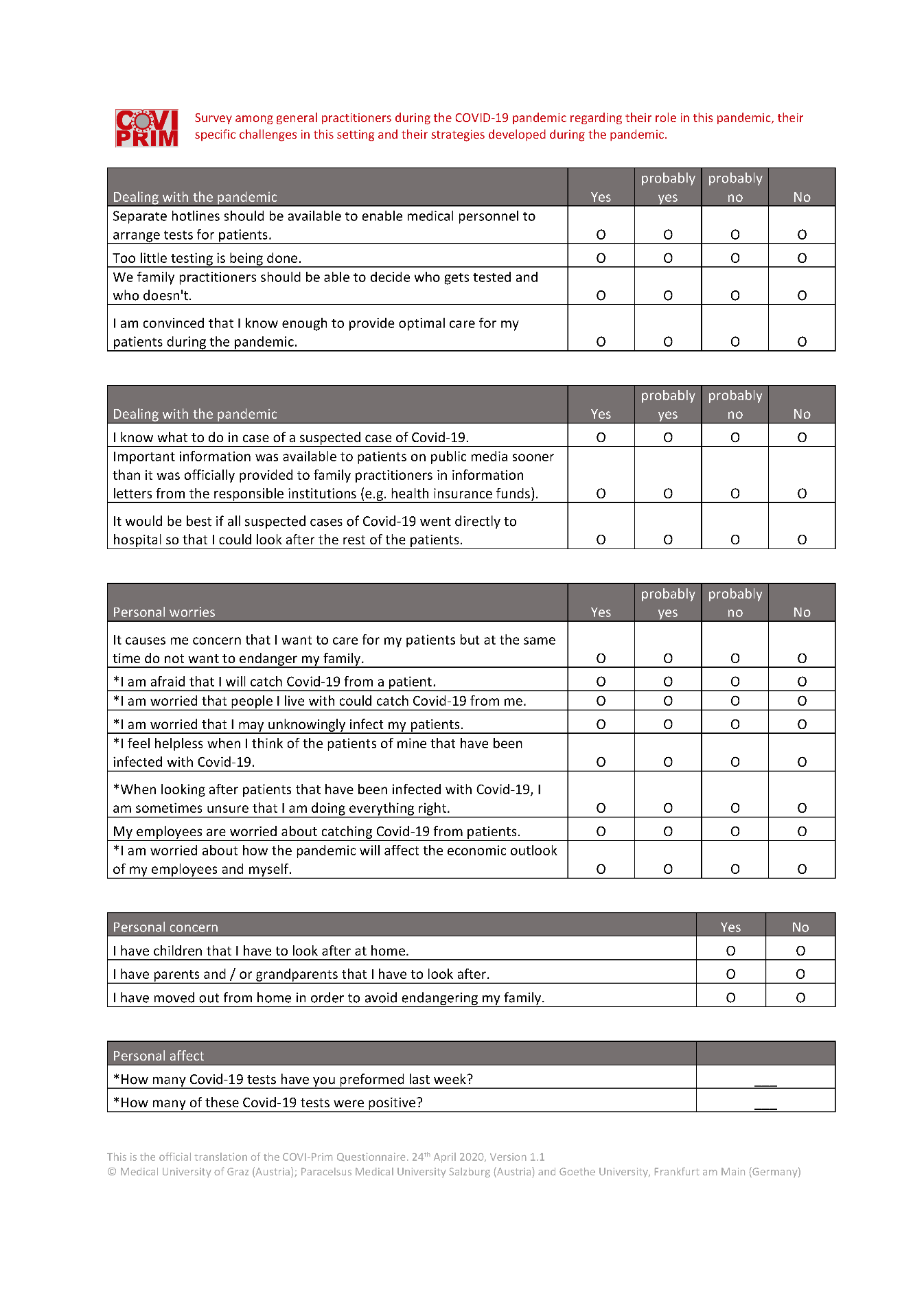

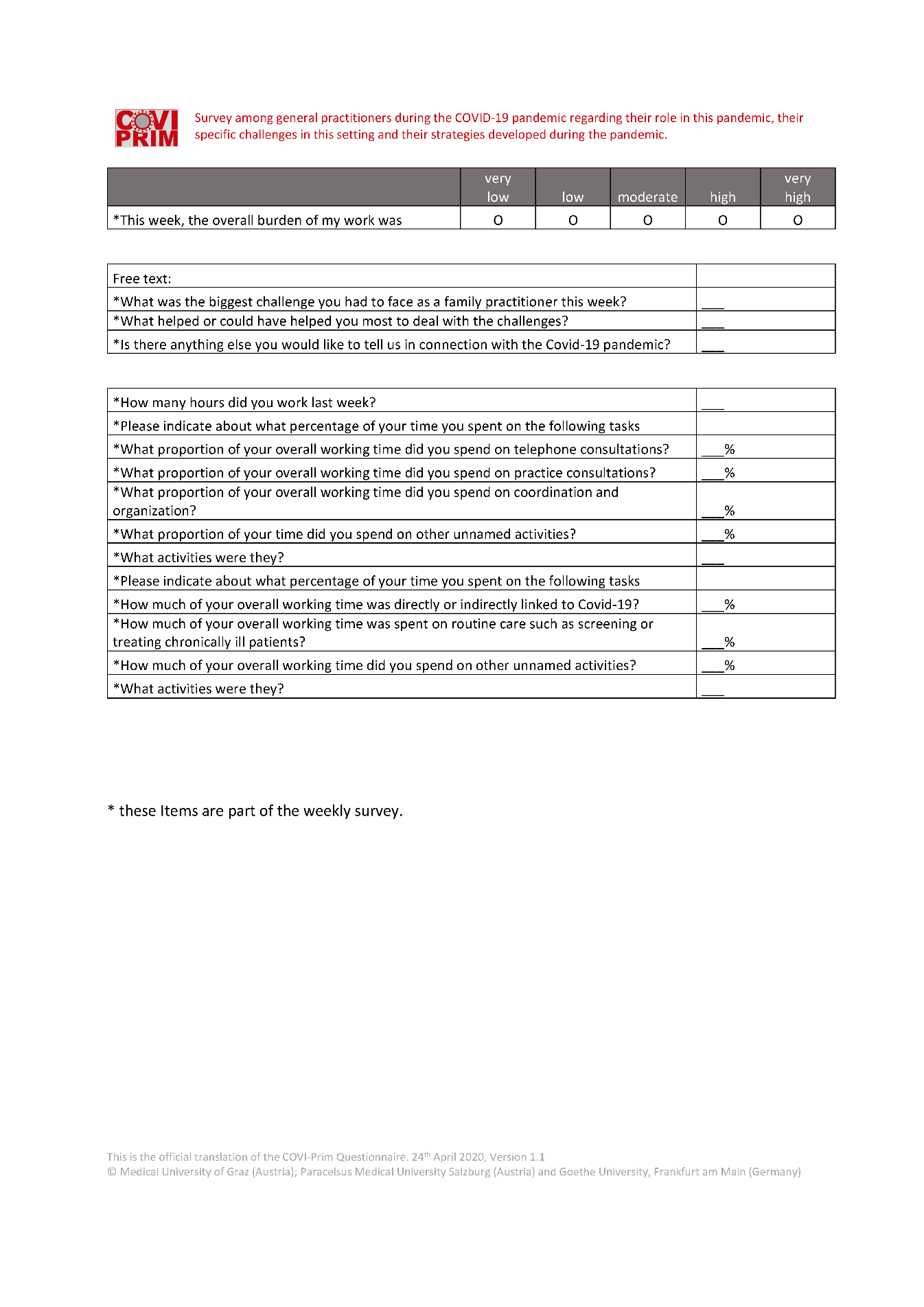


**Supplemental Digital Appendix 3.** Distribution of responses

|  |  | no | | probably  no | | probably  yes | yes |
| --- | --- | --- | --- | --- | --- | --- | --- |
|  |  |  |  |  |  |  |  |
| **Perception of risk** | |  | |  | |  |  |
|  | I am worried that people I live with could catch Covid-19 from me. | 16% | | 27% | | 32% | 25% |
|  | I am afraid that I will catch Covid-19 from a patient. | 27% | | 38% | | 20% | 15% |
|  | It causes me concern that I want to care for my patients but at the same time do not want to endanger my family. | 19% | | 27% | | 26% | 27% |
|  | I am worried that I may unknowingly infect my patients. | 14% | | 31% | | 31% | 25% |
|  | My employees are worried about catching Covid-19 from patients. | 11% | | 38% | | 32% | 20% |
| **Provision of information to GPs** | | |  | |  |  |  |
|  | I received guidelines on how to deal with suspected cases of Covid-19 in good time. | 12% | | 27% | | 41% | 20% |
|  | The guidelines on how to deal with suspected cases of Covid-19 were sufficiently detailed. | 11% | | 27% | | 42% | 20% |
|  | At the beginning of the Covid-19 pandemic, I received sufficient information from public bodies | 34% | | 35% | | 22% | 9% |
|  | At the beginning of the Covid-19 pandemic, I had sufficient information on how to deal with suspected cases. | 24% | | 29% | | 32% | 15% |
|  | My employees and I were easily able to contact the responsible health care authorities. | 29% | | 30% | | 27% | 13% |
|  | Important information was available to patients on public media sooner than it was officially provided to family practitioners in information letters from the responsible institutions (e.g. health insurance funds). | 9% | | 23% | | 30% | 39% |
| **Preparedness for a pandemic** | | |  | |  |  |  |
|  | At the beginning of the Covid-19 pandemic, I had enough protective equipment on hand. | 70% | | 16% | | 9% | 4% |
|  | My practice was well prepared for the Covid-19 pandemic. | 40% | | 34% | | 20% | 6% |
|  | At the beginning of the Covid-19 pandemic, I knew where I could get hold of protective equipment. | 52% | | 24% | | 15% | 10% |
|  | At the beginning of the Covid-19 pandemic, I had sufficient information on how much equipment I need. | 59% | | 30% | | 7% | 4% |
|  | Currently I have enough personal protective equipment. | 20% | | 24% | | 32% | 24% |
| **Self-confidence** | | |  | |  |  |  |
|  | I am convinced that I know enough to provide optimal care for my patients during the pandemic. | 3% | | 15% | | 53% | 29% |
|  | I know what to do in case of a suspected case of Covid-19. | 0% | | 1% | | 21% | 78% |
|  | When looking after patients that have been infected with Covid-19, I am sometimes unsure that I am doing everything right. | 27% | | 40% | | 25% | 7% |
| **Testing suspected cases** | | |  | |  |  |  |
|  | Too little testing is being done. | 12% | | 20% | | 24% | 45% |
|  | At the beginning of the Covid-19 pandemic I had adequate access to tests (either conducted them myself. or could arrange them). | 52% | | 17% | | 15% | 16% |
|  | It would be best if all suspected cases of Covid-19 went directly to hospital so that I could look after the rest of the patients. | 53% | | 28% | | 11% | 8% |
|  | Separate hotlines should be available to enable medical personnel to arrange tests for patients. | 8% | | 7% | | 18% | 67% |
|  | We family practitioners should be able to decide who gets tested and who doesn't. | 3% | | 6% | | 26% | 65% |
| **Decrease in number of patient contacts** | | |  | |  |  |  |
|  | I have less to do at the moment because many patients are not currently coming to the practice. | 15% | | 15% | | 32% | 38% |
|  | I have to look after more patients because other health care services (specialists. hospitals) are less available. | 37% | | 27% | | 18% | 18% |
|  | I have less contact to patients as a result of the pandemic. | 4% | | 7% | | 20% | 69% |
|  | I am currently treating patients that I would normally refer to specialists or to hospital. | 26% | | 22% | | 35% | 18% |
| **Efforts to control the spread of the disease** | | |  | |  |  |  |
|  | I do not currently treat patients with mild illnesses that are not linked to suspected cases of Covid-19 in my practice, and attend to them by phone or online. | 8% | | 8% | | 34% | 50% |
|  | If possible, I, or one of my employees, tries to gain enough information from patients by phone in order to know whether we are dealing with a suspected case of Covid-19. | 1% | | 1% | | 15% | 83% |
|  | I use various digital channels (e.g. e-mail, WhatsApp) to share information with my colleagues so that we can support each other in the current situation. | 6% | | 12% | | 27% | 55% |
|  | I have taken precautions to ensure that suspected cases do not come into contact with other patients in my practice (e.g. separate waiting rooms, appointments at different times). | 1% | | 1% | | 13% | 84% |
|  | I contact patients that are quarantined at home in order to monitor the progression of the disease. | 22% | | 13% | | 22% | 42% |
|  | I avoid touching patients when examining them. | 28% | | 25% | | 33% | 14% |
|  | Before a patient enters my practice, he or she is screened for possible symptoms (e.g. temperature measurement). | 25% | | 13% | | 22% | 39% |
| **Protection of staff** | | |  | |  |  |  |
|  | I have had to send employees home because we had too little protective equipment. | 75% | | 12% | | 6% | 7% |
|  | Some employees in my practice have ceased working since the outbreak of the Covid-19 pandemic because they belong to a vulnerable group (e.g. pregnant women, older employees). | 76% | | 5% | | 4% | 16% |
|  | I found it difficult to provide adequate information to my practice team without worrying them. | 50% | | 30% | | 15% | 5% |
